# Intact word processing in developmental prosopagnosia

**DOI:** 10.1038/s41598-017-01917-8

**Published:** 2017-05-10

**Authors:** Edwin J. Burns, Rachel J. Bennetts, Sarah Bate, Victoria C. Wright, Christoph T. Weidemann, Jeremy J. Tree

**Affiliations:** 1 Nanyang Technological University, 50 Nanyang Ave, Singapore, 639798 Singapore; 2 Bournemouth University, Fern Barrow, Poole BH12 5BB UK; 3 Aberystwyth University, Aberystwyth, UK; 4 Swansea University, Swansea University to Singleton Park, Sketty, Swansea SA2 8PP UK; 5 University of Pennsylvania, Philadelphia, Pennsylvania 19104 USA

## Abstract

A wealth of evidence from behavioural, neuropsychological and neuroimaging research supports the view that face recognition is reliant upon a domain-specific network that does not process words. In contrast, the recent many-to-many model of visual recognition posits that brain areas involved in word and face recognition are functionally integrated. Developmental prosopagnosia (DP) is characterised by severe deficits in the recognition of faces, which the many-to-many model predicts should negatively affect word recognition. Alternatively, domain-specific accounts suggest that impairments in face and word processing need not go hand in hand. To test these possibilities, we ran a battery of 7 tasks examining word processing in a group of DP cases and controls. One of our prosopagnosia cases exhibited a severe reading impairment with delayed response times during reading aloud tasks, but not lexical decision tasks. Overall, however, we found no evidence of global word processing deficits in DP, consistent with a dissociation account for face and word processing.

## Introduction

The recent many-to-many model of visual recognition proposes that specialised brain regions for the processing of faces and words are functionally integrated^1–3^; for example, areas specialised to recognise faces will also, to a lesser extent, contribute towards the recognition of words. The many-to-many model predicts that as a group, those with deficits in one area (e.g., face processing) should also show deficits in the other^4^ (e.g., word processing). Evidence for this view comes from patients with acquired prosopagnosia (AP), a disorder characterised by an inability to recognise faces following some form of trauma to the brain regions specialised for face processing; these cases have been shown to exhibit subtle word processing deficits^3^. Furthermore, individuals with alexia, a disorder associated with word processing deficits after damage to the brain areas specialised for processing words (typically the visual word form area: VWFA), have also been found to exhibit signs of face recognition impairment^3, 5^. Taken together, these findings give *prima facie* support to the many-to-many model’s proposal that word and face recognition are functionally integrated.

In general, however, evidence of associated deficits is not as compelling as evidence of a dissociation^6^. Numerous studies have shown AP and alexia cases, with unilateral damage, to be spared in their respective word and face processing abilities^7–11^. It has been suggested that the discrepancy in these results where a dissociation was found between word and face processing^7–11^, and other work that identified associative deficits between the two domains^3^, is due to the latter’s testing of AP cases that also suffered from object recognition impairments: these cases were likely impaired at an earlier stage of visual processing, or had damage to cortical areas that not only processed faces, but also contributed towards the recognition of words. The obvious conclusion from these results is that face and word recognition are reliant upon specialised processes that do not overlap.

Prosopagnosia can also be developmental (DP) in nature, occurring in individuals with no history of brain damage^12–14^. DP cases have been shown to exhibit reduced matter density and abnormal neural responses to faces throughout the brain’s face processing regions^15–19^. Typically, the Warrington Recognition Memory Test for Words^20^ has shown no evidence of word processing impairment in DP^21–23^, although it only comprises a single study-test cycle and thus may be too crude to detect subtle reading impairments. More recently it has been shown that DP cases are apparently unimpaired when reading aloud words of various lengths^24, 25^ and single letters^24^. These studies, however, comprised basic reading tasks which did not fully test word processing under a broad set of linguistic and perceptual demands.

Alexia cases exhibit abnormally slower reading latencies as word length increases, otherwise known as the word length effect (WLE)^26, 27^. However, these impairments are directly linked to damage in the VWFA and the confusability of a word’s constituent letters, that is, how perceptually similar (confusable) each letter in the word is to other letters in the alphabet^26^. For example, O is highly confusable due its similarity with C, G and Q, by contrast, X is low in confusability because of its dissimilarity to other letters^28^. When a word’s summed confusability is controlled for across words of different lengths, the WLE is abolished in alexia^26^. This suggests that alexia cases only exhibit abnormal WLEs due to the increasing confusability of a word’s constituent letters, rather than its actual length per se.

In this respect, it is maybe not surprising that DP cases evinced neurotypical reading abilities in recent studies where confusability was not controlled for^24, 25^; those with alexia only exhibit an abnormal WLE as confusability increases with increasing word length. This fact suggests the need for DP cases to be thoroughly tested on a battery of tasks where confusability is carefully controlled for. If alexia and DP cases share similar deficits in their early perceptual processing of faces and words, then those with DP should show similarly elevated WLEs when the sum confusability of a word increases with word length. Conversely, we should also see those with DP exhibit neurotypical WLEs when asked to read aloud words where sum confusability is held constant as word length increases.

In addition to reading aloud, lexical decision tasks, where participants are asked to quickly decide whether a presented string of letters constitutes a valid word or not, are a popular tool to test word recognition^29^. While neuropsychological evidence has shown that damage to the VWFA impairs reading aloud, lexical decision making is spared^30^, suggesting a dissociation between these two tasks. However, despite reading words aloud and alexia being directly linked to the VWFA, neuroimaging research has suggested that reading relies more on the dorsal pathway, whereas lexical decisions are associated with a stronger involvement of the occipito-temporal cortex^31–33^ which includes many of the face related cortical regions. A case could therefore be made that lexical decision tasks, rather than simple reading aloud tasks, might be better suited to testing the many-to-many model’s predictions of common word and face processing deficits in DP.

Similarly, DP cases are characterised by their very inability to retrieve confirmation that a face has been encountered before. We hypothesise that performance in lexical decision tasks, rather than naming tasks, might be more diagnostic of the common difficulties DP cases experience when judging facial identity. When participants see a word during a lexical decision task, they need to access the semantic memory system which stores facts about the world to confirm that they know that this word is a word^34^. Recognition memory models typically posit that recognition works the same way for different types of stimuli, with words and faces both able to elicit a familiarity signal on which a recognition decision is based^35^. There are a series of stages at which this type of recognition can fail for face stimuli in DP. In some cases, those with DP may fail to match the presented face to a previously stored representation due to poor perceptual processing of the face’s attributes. Other DP cases, however, are thought to be successful in perceptually activating this stored representation of the presented face^36^. Instead, this success in perceptual processing somehow fails to connect downstream to the semantic memory store to confirm familiarity or to episodic memory where information such as when and where the face was previously encountered is stored. In this respect, if the face recognition system is integrated with word recognition, then we should expect to see those with DP exhibiting similar failures, either through mistakenly judging the lexicality of a visually presented word or non-word, or being slower in confirming word familiarity due to degraded perceptual processing. It should be noted that individuals with DP are generally able to confirm that a celebrity’s name is known to them. For example, after a famous faces test the experimenter will check whether the DP case has failed to recognise a particular face because of their face recognition problems, or simply because they do not know who the celebrity is. While this may indicate that DP cases are unimpaired at processing the familiarity of non-face stimuli, no study has yet confirmed this fact with a lexical decision task.

Confusability and word length place distinct perceptual demands upon the visual recognition system, however, this system can also be tested in its ability to process words of changing linguistic complexity. For example, the mere frequency of a word appearing in written language can crudely index one’s level of visual experience with that word. If DP is associated with deficits in their sensitivity to experience, then such deficits should not only impact their ability to identify famous faces, but also impair performance on word processing tasks where word frequency is varied. Similarly, the age at which one acquires a word has also been shown to affect reading performance^37^. Age of Acquisition (AoA), however, is linked to word frequency, and both variables should therefore be examined jointly. Finally, a word’s orthographic neighbourhood is comprised of all other words that can be derived by changing one of its constituent letters (the size of a word’s neighbourhood is denoted by N)^38^; for example, lob has the orthographic neighbours mob, log, lot and lab. Intriguingly, activity in the brain’s right hemisphere, which exhibits many of the neural abnormalities in DP^15–19^, appears to be sensitive to N^39^. Under the assumptions of the many-to-many model, one might therefore expect any linguistic deficits in DP to vary with N.

We tested the many-to-many model’s account of visual recognition by examining the performance of a group of DP cases on a comprehensive battery of 7 behavioural word recognition experiments. We label tasks where we vary word length across conditions as testing the role of perceptual information in word processing due to the fact that such a manipulation varies the physical length of our stimuli between trials. By contrast, any task that maintains the physical length of words while varying linguistic properties, such as frequency or AoA, will be labelled as testing the processing of linguistic information. We should add a caveat, however, that this classification is rather crude and is only meant to facilitate discussion of the different tasks. While the many-to-many model broadly predicts word processing impairments in prosopagnosia, it may be the case that these impairments only manifest themselves when demands are placed upon perceptual, rather than linguistic, processing. If DP cases were also impaired in linguistic processing, then it might indicate a much more basic, low level visual problem where words and faces are processed prior to functionally specialised regions. We therefore wanted to examine whether this was the case across perceptual and linguistic tasks. Each set of tasks consisted of one lexical decision task and a number of word reading tests.

## Methods

### Participants

The 11 DP cases that participated in the behavioural tasks were aged 20–73 years old (Mean = 41.55 years, 3 males). The 37 controls comprised of 2 groups: a younger group of 18 participants aged between 20–33 years (Mean = 23 years, 6 males) and an older group of 19 participants ranging from 56–77 years (Mean = 66 years, 7 male) to be roughly comparable to DP cases aged 32 years and younger or 52 years and older respectively. Due to the small numbers in each group, they were collapsed together for our analyses. All participants had normal or corrected to normal vision and were native English speakers. All controls and DP cases were either studying at, or had completed, university education. None of the controls reported difficulties in recognising faces, a fundamental criterion for prosopagnosia, and none of the participants had dyslexia. It should be noted that due to time constraints, not all DP cases completed all 7 behavioural tasks but their data is still included where possible. The study was given ethical approval by the Swansea University Research Ethics Committee. All methods were carried out in accordance with approved guidelines and required informed consent to be obtained from all participants.

**Figure 1:**
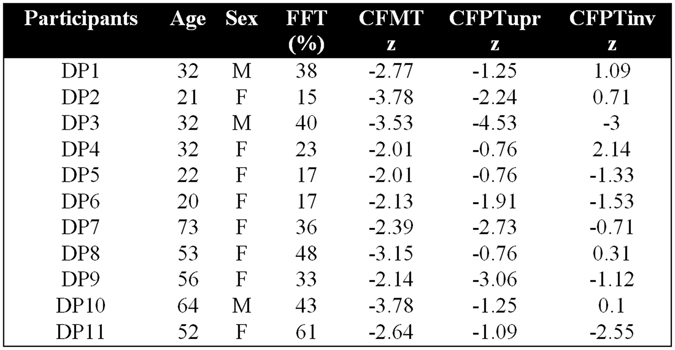
Neuropsychological testing results of the 11 DP cases that participated in the experiments. Columns indicate: Famous Faces Test (FFT), Cambridge Face Memory Test (CFMT), Cambridge Face Perception Test upright and inverted (CFPTupr and CFPTinv).

Figure 1 lists the DP cases that participated in the experiments and their neuropsychological tests of face processing impairment, which included: a shortened Famous Faces Test^40^ (FFT), the Cambridge Face Memory^41^ (CFMT), and the Cambridge Face Perception Test^42^ (CFPT), with further details found in the citations. We collected data for the shortened FFT from 164 participants (101 female) to ascertain normative means and SDs for the general population in the local geographical area (M = 94.6%, SD = 6.23). Normative scores for the CFMT and CFPT were taken from the cited literature. As can be seen from Fig. 1, all of our DP participants scored more than 2 SD below the control mean on the FFT and CFMT, with 4 showing impaired performance on the CFPT. As with previous DP research^43–45^, our criteria for identifying DP cases required impairment on both the CFMT and FFT.

### General Procedure

The seven experiments were completed in a random sequence for each participant. We analysed our data using mixed model ANOVAs, the purpose of which was to test the prediction that individuals with prosopagnosia should, as a population, exhibit word processing deficits^4^. To this end, we only report main effects or interactions involving the factor Group (controls vs. DP cases), with any follow up comparisons Bonferroni corrected. All response times were for correct responses and all group analyses two-tailed. Bayesian analyses were also performed to test the weight of evidence for the null hypothesis (Supplementary Information). Slope values for the word length effect^27^ were calculated by regressing the response times and errors, with individual DP cases’ WLEs reported in the Supplementary Information. Additionally we used the Crawford’s t-test^46^ to detect any abnormalities in individual DP cases’ performance. As we were testing the many-to-many model’s prediction that DP cases would exhibit global deficits in word recognition, we used a one-tailed test with 18 degrees of freedom to produce a critical t-value of 1.737 for the older DP cases; any individual with a t-value above this score will be identified as impaired. The critical t-value for the younger group with 17 degrees of freedom was 1.743. Any variables (e.g., bigram frequency) that were matched across conditions on any given task were confirmed as not being statistically different from one another. All word lists are provided on Scientific Reports’ website.

### Impact of Perceptual Information (Word Length)

#### Lexical Decision: Length (word confusability not controlled)

Lexical decision tasks should reveal any difficulties DP cases may have in confirming word familiarity under perceptually demanding conditions of varying word length. Stimuli comprised 120 words and non-words. The 120 words consisted of 3 groups of 3-, 5- or 7-letters in length. Groups were matched for CELEX frequency, AoA (Bristol Norms) and bigram frequency. Mean bigram frequency merely means the frequency with which any pairs of adjacent letters found in a word occur within the printed English language. It was not possible to control for N across the 3 different letter length groups due to the inverse relationship between N and word length: 3-letter words avg. 13 neighbours, 5-letter words avg. 2.25 neighbours, 7-letter words avg. 0.2 neighbours. The 160 non-words were taken from the ARC Non-Word Database^47^. Non-words were pseudowords matched with the respective word stimuli for string length, orthographic neighbours, and bigram frequency. Examples include treaps, grauds and guites.

Each trial began with a centrally presented black fixation cross for 2000 ms against a white background. Then one of the 160 word or 160 non-word targets was presented in black, replacing the fixation cross. Participants were required to judge as quickly and accurately as they could, whether each target was a word or non-word by pressing the appropriate response keys on a keyboard. Immediately after their response, an asterisk (*) appeared onscreen for 500 ms before the beginning of the next trial. Presentation of the stimuli was randomised and controlled using SuperLab Pro. Stimuli were presented in 24 point, lower-case Arial font. Prior to the experiment, participants were required to complete 12 practice trials (6 words and 6 non-words).

#### Reading Aloud: Length (word confusability not controlled)

Alexia cases are impaired when asked to read aloud words of different lengths where confusability is not controlled for. To test whether DP cases exhibit similar impairment, we designed the present task to mimic such conditions. Word stimuli were the same as in the previous task, however, the non-words were not used; all of our reading aloud stimuli lists comprised real words alone. Each trial was exactly the same as described for the previous task apart from the following details: instead of responding word or non-word by pressing response keys, participants were required to read the word aloud when it was presented on the screen. The targets remained on the screen until the participant responded. Vocal responses were detected using an SV-1 voice key (Cedrus Software). Due to the fact that the voice key could be triggered by any sound, participants’ responses were also checked for accuracy from separate recordings using a digital voice recorder. As for the lexical decision task, participants initially completed 6 practice trials.

#### Reading Aloud: Length (sum confusability maintained across words)

Alexia cases are spared when reading words of different lengths where the sum confusability of all words is maintained. To examine whether DP cases exhibit similar performance, we controlled the sum confusability of all words in this task so confusability was the same for each word regardless of word length. Stimuli were comprised of 120 words taken from prior work on summed confusability so that any abnormal performance by our DP cases could be interpreted with respect to their alexia cases^26^. Words were matched on N, summed letter confusability and frequency while varying word length, with the 120 items comprising equal numbers of 5-, 6-, and 7-letter long words. The procedure was the same as the previous reading aloud task. Participants had to complete 6 practice trials prior to the experiment.

#### Reading Aloud: Length (average letter confusability maintained across words)

As mentioned, alexia cases are impaired when confusability increases across words of different lengths. We decided to better control this variable than in the second length task by maintaining the average letter confusability across words of different lengths. This will have the effect of increasing the average word confusability in a linear fashion as word length increases. If DP cases have similar difficulties in reading as those with alexia, then they should exhibit elevated WLE when attempting to read words in this condition. Stimuli comprised of 120 words again taken from prior work^26^, and were matched on N, average letter confusability, and frequency. Length was varied with equal numbers of our 120 stimuli comprised of 5-, 6-, and 7-letter long words. The procedure was the same as for the previous reading aloud task and included 6 practice trials.

### Impact of Linguistic Information

#### Lexical Decision: Frequency x Age of Acquisition (AoA)

Word frequency crudely indexes our visual experience with different words. As AoA influences performance where frequency is varied, we tested participants across words that varied in AoA too. Stimuli comprised 160 words and 160 non-words. The 160 words were divided into four orthogonal conditions according to AoA (early/late) and frequency of use (high/low): half of the words were early acquired (Mean = 5.37 years of age, earliest word = 3.7 years of age, latest word = 8.3 years of age), with the remainder acquired late (Mean = 9.29 years of age, earliest word = 6.7 years of age, latest word = 12.6 years of age; Bristol Norms^48^). While there was some overlap between the highest and lowest AoA groups, this was necessary to still enable us to have distinct high and low frequency conditions. Within each condition (early/late AoA), half were high-frequency words and the remaining half were low-frequency. High frequency words had a word frequency score of >240 per million whereas low frequency words were <30 per million (CELEX database^49^). Words were matched across all groups for length (in letters), number of orthographic neighbours (N) and mean bigram frequency. The 160 non-words were taken from the ARC Non-Word Database^47^. Non-words were also assigned into four groups and matched with the respective word stimuli for string length, orthographic neighbours, and bigram frequency. The procedure was exactly the same as the previous lexical decision task.

#### Reading Aloud: Frequency x AoA

We used the word, but not non-word, stimuli from the above lexical decision task crossing word frequency with AoA in a reading aloud task using the same procedures as described for previous word naming tasks.

#### Reading Aloud: N Confusability

N has been shown to modulate reading performance in alexia cases when letter confusability is varied^50^. We therefore examined reading performance across different levels of N and letter confusability. It should be noted that this task does place considerable perceptual demands upon the visual recognition system, so may not be as exclusively testing linguistic processing as our previous linguistic tasks. Stimuli consisted of 200, 4-letter long words taken from prior work on alexia cases so that our results could be comparable if our DP cases were abnormal^50^. The words were varied by letter confusability and N. The words were split into 4 groups: 50 high confusability high N, 50 high confusability low N, 50 low confusability high N, and 50 low confusability low N. The cutoffs were: low N < 5, high N > 8, low confusability <0.45, and high confusability >0.53. Participants had to complete 10 practice trials prior to the main task. Procedure was the same as previous reading aloud tasks.

## Results

### Impact of Perceptual Information

#### Lexical Decision: Length (word confusability not controlled)

**Figure 2:**
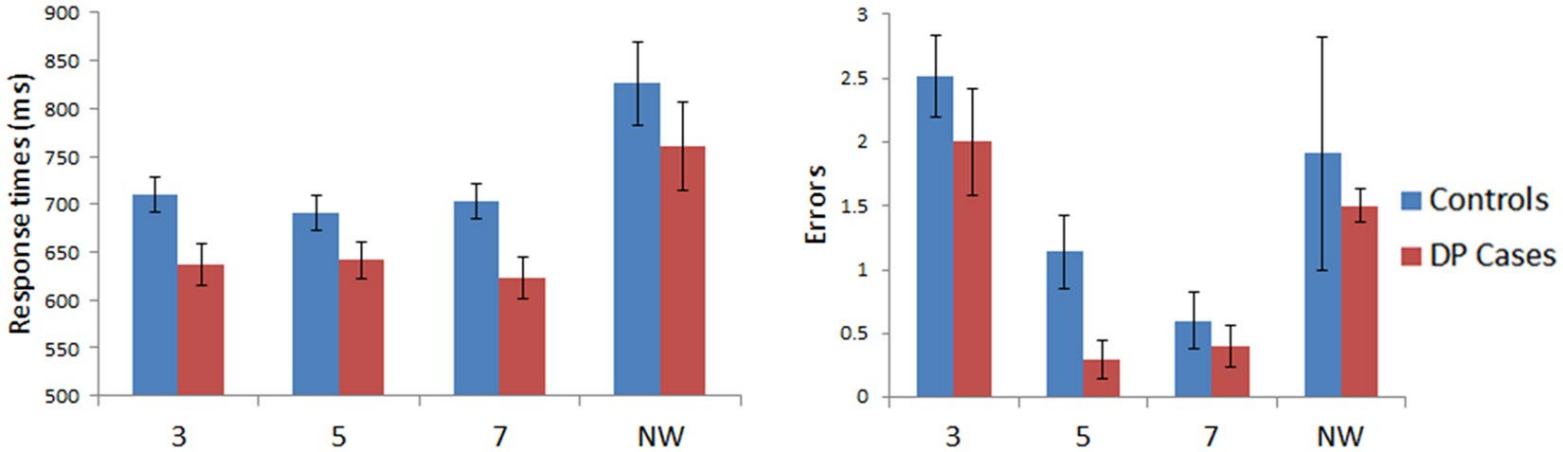
Results for Lexical Decision: Length (word confusability not controlled). Data for words and non-words (NW) are shown in left panel (response time) and right panel (number of errors) for control and DP groups. Error bars indicate ±SEM. The three word conditions are words comprised of 3-, 5-, or 7-letters. As there were 3 times more items in the NW condition, the NW errors were divided by 3 to make their rates displayed in the graph proportionally comparable to the word conditions.

Figure 2 presents the results for the lexical decision task where word length was varied. To test for any possible effects of lexicality between the groups, response times were subjected to a mixed model ANOVA with Stimuli (words vs. non-words) as a within subject factor and with Group (controls vs. DP) as a between subject factor. No significant effect for the factor Group [F (1, 45) = 1.47, MSE = 68909, p = 0.23] nor any significant Group x Stimuli interaction was found [F (1, 45) = 0.001, MSE = 22, p = 0.98]. A similar ANOVA performed on the errors also revealed no effect for Group [F (1, 45) = 0.75, MSE = 12, p = 0.39], nor any Group x Stimuli [F (1, 45) = 0.15, MSE = 2, p = 0.7] interaction.

To examine any possible differences between response times across length of word stimuli, an ANOVA was performed with factors of Length (3-, 5- and 7-letters) as a within subject factor and Group (controls vs. DP) as between subject factors. No significant Group [F (1, 45) = 3.6, MSE = 107057, p = 0.064] effect was found, nor any significant Length x Group interaction [F (2, 90) = 1.76, MSE = 2039, p = 0.18]. Between group comparisons on the WLE slopes also indicated that there were no significant response time (DP: *M* = −16.44 ms/letter; Controls: *M* = 2.58 ms/letter, [t(45) = 1.32, p = 0.2]) or error related (DP: *M* = −.75 errors/letter; Controls: *M* = −1.23 errors/letter, [t(45) = 1.09, p = 0.28]) WLE differences between the groups. The same ANOVA performed on the errors yielded no significant effect of Group [F (1, 45) = 1.15, MSE = 6.25, p = 0.29] nor a Group x Length interaction [F (2, 90) = 0.79, MSE = 0.81, p = 0.46]. In summary, our analyses revealed that those with DP do not exhibit any deficits in lexical decisions as word length is varied.

#### Reading Aloud: Length (word confusability not controlled)

**Figure 3:**
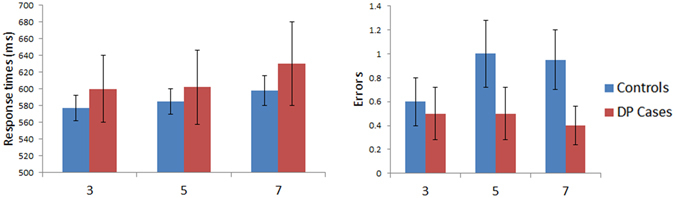
Results for Reading Aloud: Length (word confusability not controlled). Data are shown in left panel (response time) and right panel (number of errors) for control and DP groups. Error bars display ±SEM. The three conditions are words comprised of 3-, 5-, and 7-letters.

Figure 3 displays the results for the reading aloud task where word length was varied. A mixed model ANOVA was performed on the response times with Length (3-, 5- and 7-letters) as a within subject factor and with Group (controls vs. DP) as a between subject factor. No significant effect was found for Group [F (1, 45) = 0.41, MSE = 13187, p = 0.53] nor any significant Group x Length interaction [F (2, 90) = 0.39, MSE = 421, p = 0.68] either. A mixed model ANOVA performed on the errors revealed no main effect for Group [F (1, 45) = 0.8, MSE = 3.41, p = 0.38] nor any significant Group x Length interaction [F (2, 90) = 0.72, MSE = 0.49, p = 0.49]. Independent samples t-tests on the WLE slopes for the response times (DP: *M* = 14.64 ms/letter; Controls: *M* = 10.79 ms/letter, [t(45) = 0.38, p = 0.7]) and errors (DP: *M* = −0.05 errors/letter; Controls: *M* = 0.18 errors/letter, [t(45) = 1.02, p = 0.31]) found no significant WLE differences between the groups. In summary, the DP group exhibited no impairment in their performance when reading words of different lengths.

#### Reading Aloud: Length (sum confusability of letters maintained across words)

**Figure 4:**
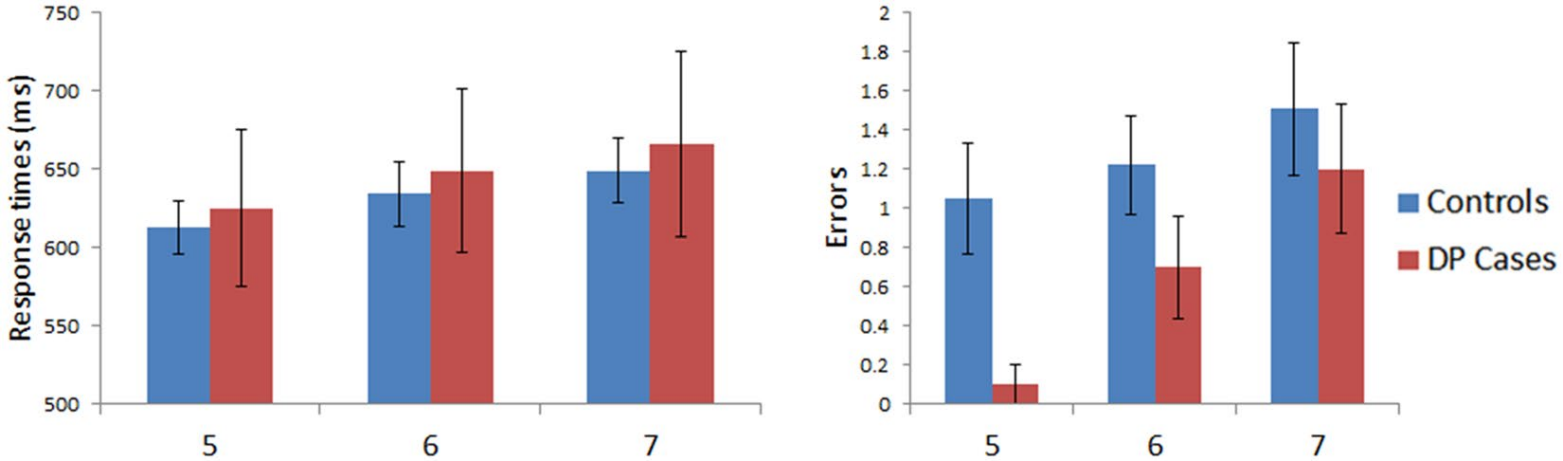
Results for Reading Aloud: Length (sum confusability maintained across words). Data are shown in left panel (response time) and right panel (number of errors) for control and DP groups. Error bars show ±SEM. The three conditions are words comprised of 5-, 6-, or 7-letters with sum confusability controlled for across all words.

Figure 4 displays the results for the reading task where sum confusability was kept constant across varying word lengths. A mixed model ANOVA was performed on the response times with Length (5-, 6- and 7-letters) as a within subject factor and with Group (controls vs. DP) as a between subject factor. We found no significant Group effect [F (1, 45) = 0.11, MSE = 5343, p = 0.75] nor any Group x Length [F (2, 90) = 0.07, MSE = 62, p = 0.93] interaction. A similar ANOVA performed on the errors also produced no effect of Group [F (1, 45) = 1.35, MSE = 8.35, p = 0.25] nor any Group x Length [F (2, 90) = 1.01, MSE = 0.84, p = 0.37] interaction. Between group comparisons on the slopes showed no significant differences between the groups in their response time (DP: *M* = 20.33 ms/letter; Controls: *M* = 17.58 ms/letter, [t(45) = 0.31, p = 0.76]) nor error rate related (DP: *M* = 0.55 errors/letter; Controls: *M* = 0.23 errors/letter, [t(45) = 1.61, p = 0.11]) WLE. In summary, those with DP appear to have no impairment in their reading abilities across words of different lengths when controlling for sum confusability.

#### Reading Aloud: Length (average letter confusability maintained across words)

**Figure 5:**
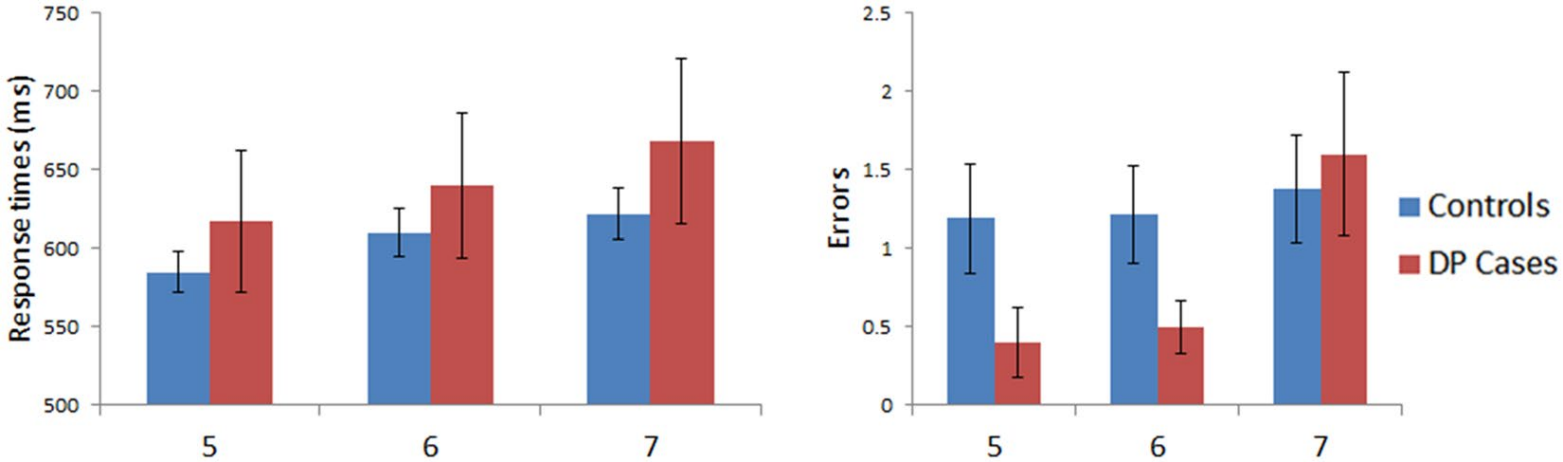
Results for Reading Aloud: Length (average letter confusability maintained across words). Data are shown in left panel (response time) and right panel (number of errors) for control and DP groups. Error bars show ±SEM. The three conditions are words comprised of 5-, 6-, or 7-letters with average confusability controlled for across all words.

Figure 5 shows the results for the reading task where average confusability was kept constant as word length was varied. A mixed model ANOVA was performed on the response times with Length (5-, 6- and 7-letters) as a within subject factor and with Group (controls vs. DP) as a between subject factor. No significant main effect of Group was found [F (1, 45) = 0.96, MSE = 30264, p = 0.33], nor any significant Group x Length interaction [F (2, 90) = 0.83, MSE = 609, p = 0.44]. A similar ANOVA performed on the errors revealed no significant main effect for Group [F (1, 45) = 0.54, MSE = 4.33, p = 0.47]. The Group x Length interaction was not significant either [F (2, 90) = 1.91, MSE = 2.5, p = 0.15]. Between group comparisons on the slope values yielded no significant differences between the groups in their response time WLE (DP: *M* = 25.41 ms/letter; Controls: *M* = 18.36 ms/letter, [t(45) = 0.95, p = 0.35]) but the DP group exhibited an abnormal trend in their error related WLE (DP: *M* = 0.6 errors/letter; Controls: *M* = 0.1 errors/letter, [t(45) = 1.84, p = 0.073]). Visual inspection of Fig. 5 shows that this was due to the DP cases evincing superior performance in the 5- and 6-letter long word conditions, but comparable performance to the controls in the 7-letter condition. This suggests no apparent abnormalities in the DP group despite their elevated WLE. Overall, the DP cases did not exhibit any deficits when reading words of different lengths where average letter confusability was kept constant, as shown by their neurotypical response times and errors made.

### Impact of Linguistic Information

#### Lexical Decision: Frequency x Age of Acquisition (AoA)

**Figure 6:**
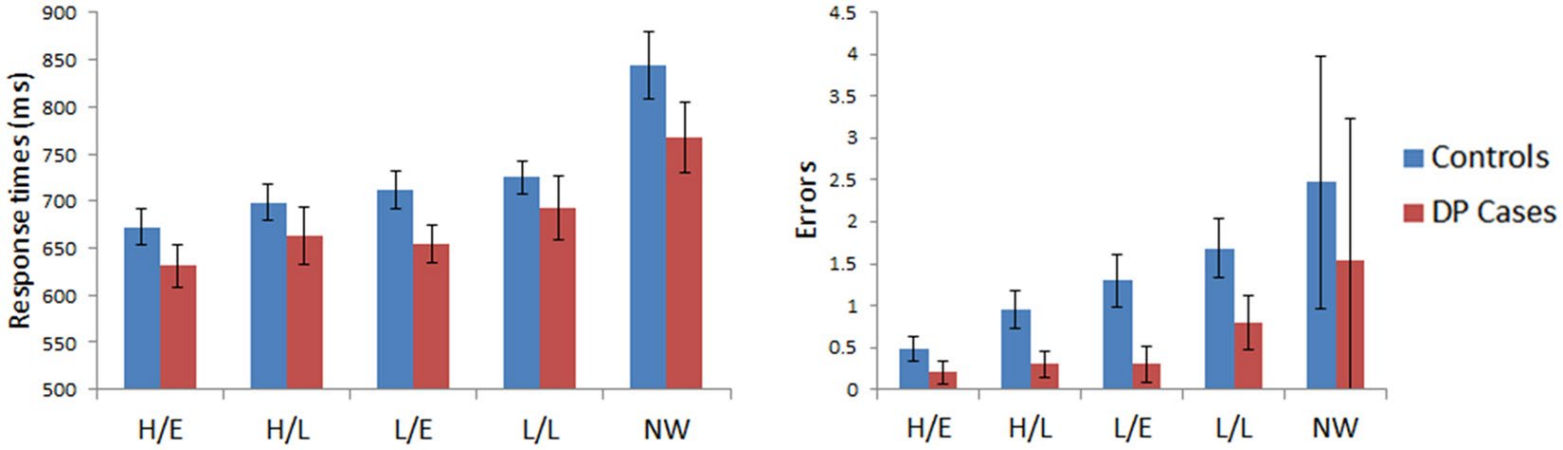
Results for Lexical Decision: Frequency x AoA. Data for words and non-words (NW) are shown in left panel (response time) and right panel (number of errors) for control and DP groups. Error bars indicate ±SEM. The four word conditions are high frequency/early acquisition (H/E), high frequency/late acquisition (H/L), low frequency/early acquisition (L/E), and low frequency/late acquisition (L/L). As there were 4 times more items in the NW condition, the NW errors were divided by 4 to make their rates displayed in the graph proportionally comparable to the word conditions.

Figure 6 presents the results for the lexical decision task that varied word frequency and age of acquisition. In order to test for any possible differences between the groups for lexicality, response times were subjected to a mixed model ANOVA with Stimuli (words vs. non-words) as a within subject factor and Group (controls vs. DP) as a between subject factor. No significant main effect for Group [F (1, 45) = 1.38, MSE = 55017, p = 0.25] nor any Group x Stimuli interaction [F (1, 45) = 0.36, MSE = 4640, p = 0.55] was found. A similar ANOVA on the errors revealed no significant effect was found for Group [F (1, 45) = 2.45, MSE = 166, p = 0.13]. As with the response times, no significant interaction involving Group was found either [F (1, 45) = 0.12, MSE = 3.48, p = 0.74]. Overall, there appears to be no impairment in DP when making judgments of lexicality.

We examined any possible differences between the two groups for word types by performing an ANOVA on the response time data, with factors of Frequency (high vs. low) and AoA (early vs. late) as within subject factors and Group (controls vs. DP) as a between subject factor. No significant effect involving Group [F (1, 45) = 1.29, MSE = 55402, p = 0.26], nor any significant Group x Frequency [F (1, 45) = 0.27, MSE = 440, p = 0.61] or Group x AoA interactions [F (1, 45) = 1.45, MSE = 1942, p = 0.24] were found. Similar analyses on the errors revealed no significant effect of Group [F (1, 45) = 2.44, MSE = 15, p = 0.13], nor any significant Group x Frequency [F (1, 45) = 1.04, MSE = 1.64, p = 0.31] or Group x AoA [F (1, 45) = 0.2, MSE = 0.14, p = 0.66] interactions. In summary, our analyses revealed no differences between the controls and DP cases in their response times or errors made on our lexical decision task when varying age of acquisition and word frequency.

#### Reading Aloud: Frequency x AoA

**Figure 7:**
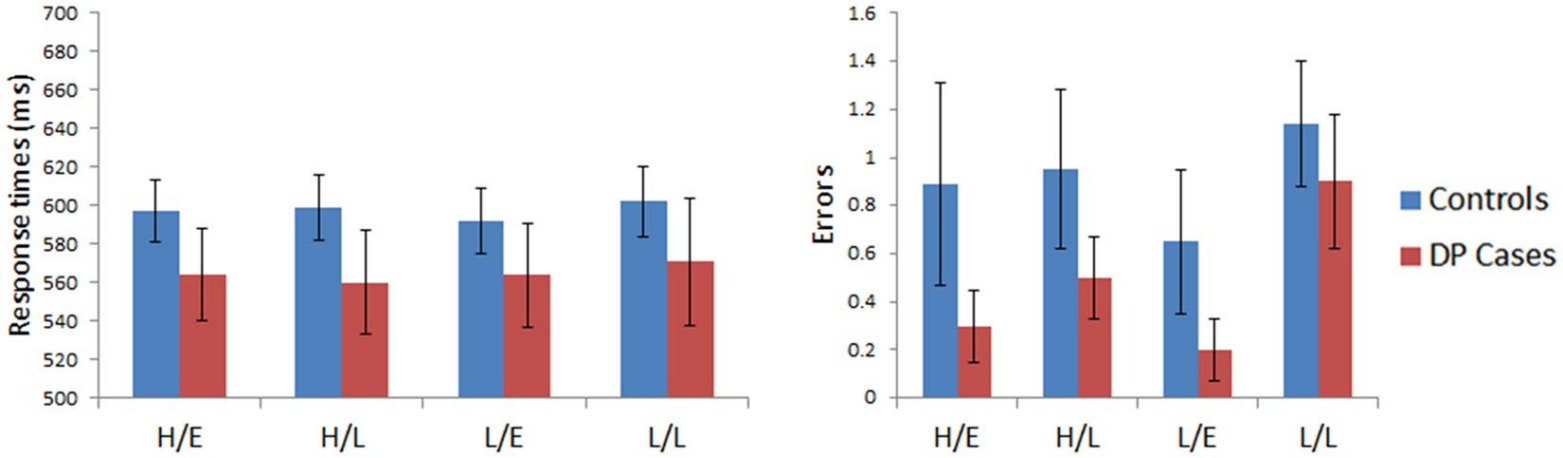
Results for Reading Aloud: Frequency x AoA. Data are shown in left panel (response time) and right panel (number of errors) for control and DP groups. Error bars indicate± SEM. The four conditions are words of high frequency/early acquisition (H/E), high frequency/late acquisition (H/L), low frequency/early acquisition (L/E), and low frequency/late acquisition (L/L).

Figure 7 presents the results for the reading aloud task that varied word frequency and age of acquisition. Response times were subjected to a mixed model ANOVA with Frequency (high vs. low) and AoA as within subject factors and with Group (controls vs. DP) as a between subject factor. We found no significant effect for Group [F (1, 45) = 0.8, MSE = 32077, p = 0.38], nor any significant Group x Frequency [F (1, 45) = 0.4, MSE = 184, p = 0.53] or Group x AoA [F (1, 45) = 0.52, MSE = 233, p = 0.48] interaction.

We also performed a mixed model ANOVA on the errors, but these analyses revealed no significant main effect for Group [F (1, 45) = 0.55, MSE = 5.8, p = 0.46] as well as no Group x Frequency [F (1, 45) = 0.44, MSE = 0.25, p = 0.51] or Group x AoA [F (1, 45) = 0.15, MSE = 0.25, p = 0.7] interactions. In summary, word reading performance for individuals with DP was similar to those of the controls when varying age of acquisition and word frequency.

#### Reading Aloud: N Confusability

**Figure 8:**
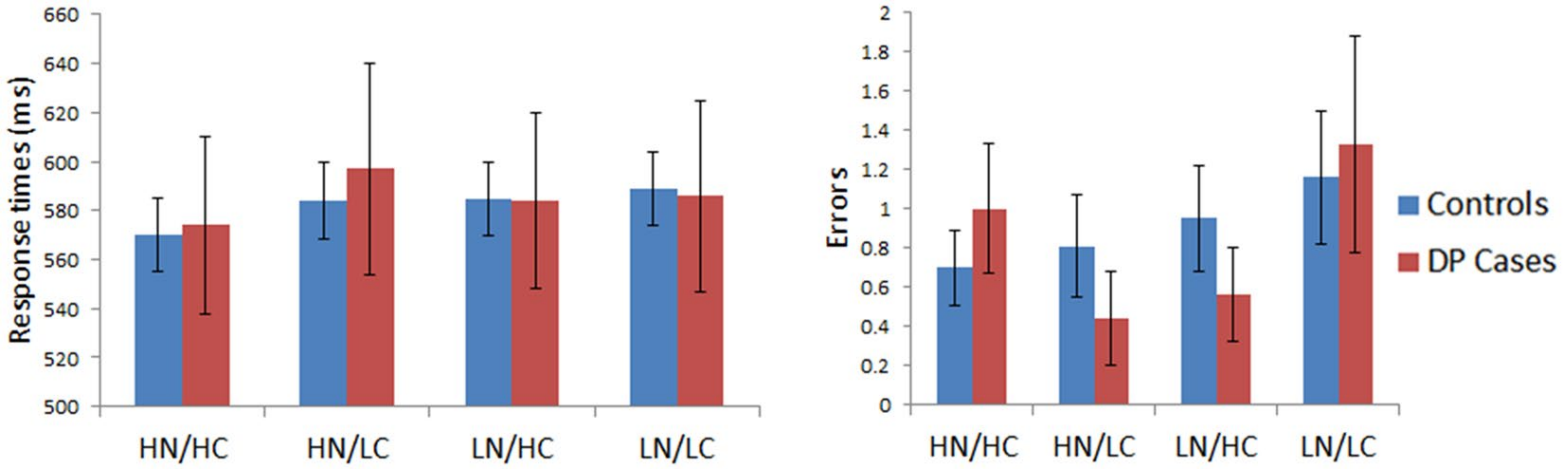
Results for Reading Aloud: N Confusability. Data are shown in left panel (response time) and right panel (number of errors) for control and DP groups. Error bars indicate ±SEM. The four conditions are words of high n/high confusability (HN/HC), high n/low confusability (HN/LC), low n/high confusability (LN/HC), and low n/low confusability (LN/LC).

Figure 8 displays the results for the reading task where N and confusability were varied. Response times were subjected to a mixed model ANOVA with N (high vs. low) and Confusability (High vs. Low) as within subject factors and with Group (controls vs. DP) as a between subject factor. No significant main effect for Group was found [F (1, 44) = 0.007, MSE = 247, p = 0.94], nor any significant Group x Confusability [F (1, 44) = 0.13, MSE = 73, p = 0.72] or Group x N [F (1, 44) = 2.34, MSE = 877, p = 0.13] interactions. A similar ANOVA performed on the errors revealed no significant main effect for Group [F (1, 44) = 0.024, MSE = 0.15, p = 0.88]. The Group x Confusability [F (1, 44) = 0.027, MSE = 0.019, p = 0.87] and Group x N [F (1, 44) = 0.029, MSE = 0.041, p = 0.87] interactions were not significant either. As in the case of previous tasks, we failed to find evidence of any deficits in DP cases’ abilities to read words that were varied by N and Confusability.

### Global Analyses of Behavioural Tasks

We were concerned that there might have been a global deficit in word recognition in DP that was not identified by our analyses on any individual task. To this end, we collapsed each task’s word conditions together to give us a mean response time for word processing on any given task. These values were then converted to z-scores to standardise measures across experiments, and subjected to a 2 × 2 × 7 mixed model ANOVA with a between subject factor of Group (controls vs. DP) and within subject factors of Experiment (1, 2, 3, 4, 5, 6 and 7) and Measure (Response Times and Errors). These analyses revealed no significant effect for Group [F (1, 43) = 1.08, MSE = 4.35, p = 0.31] nor any Group x Experiment [F(6, 258) = 1.04, MSE = 0.4, p = 0.4], Group x Measure [F(1, 43) = 0.02, MSE = 0.03, p = 0.9], or Group x Experiment x Measure [F(6, 258) = 0.43, MSE = 0.13, p = 0.86] interactions. In conclusion, those with DP exhibited no evidence of global word processing deficits.

### Performance of Individual DP Cases Impaired on a Single Condition

**Figure 9:**
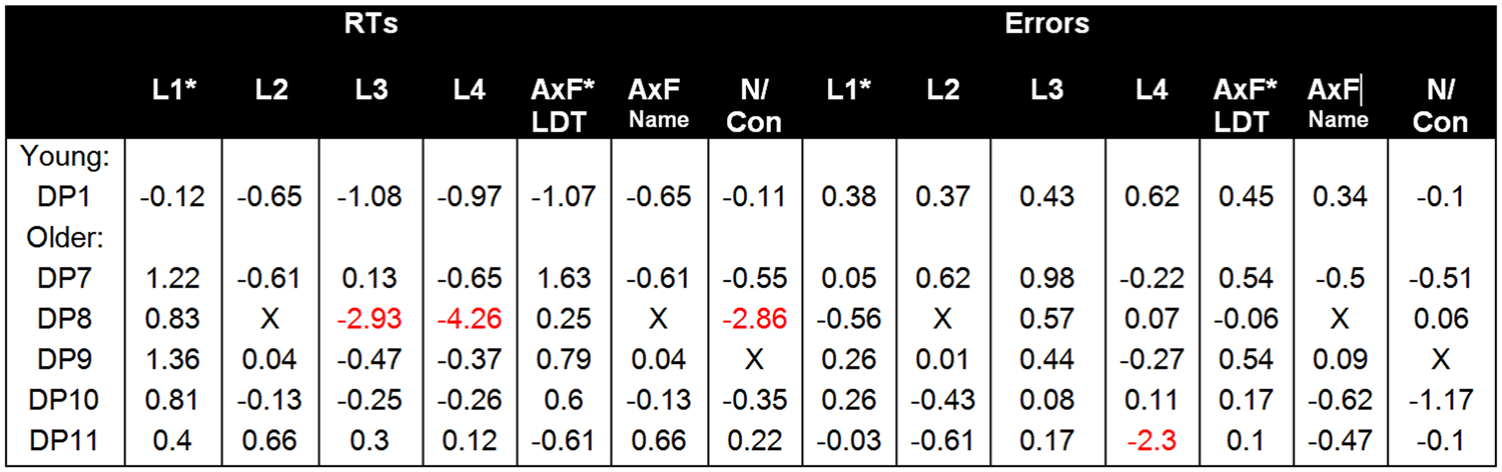
T-values of individual DP cases that were impaired on a single condition. The columns on the left show response time performance with the columns on the right showing the error rates. L1-L4 relates to the four length tasks in order as presented in our Results section. AxF LDT = Age of Acquisition × Frequency Lexical Decision task, AxF Name = Age of Acquisition × Frequency Naming task, N/Con = N × Confusability task. Asterisks highlight lexical decision tasks and Xs denote tasks that were not completed by the participant.

The many-to-many model predicts that as a group, prosopagnosia cases should exhibit word processing deficits. We found no evidence to support this suggestion anywhere in our group analyses. To better examine whether there were any consistent impairments in our individual DP cases across tasks, we present the average t-values of each DP case that was impaired in any single condition for each task in Fig. 9. DP8 was the only case that exhibited clear impairment, with abnormally slower response times apparent on all reading aloud tasks, although they were spared in their error rates. Remarkably, DP8 appeared superior to the control mean response times when making lexical judgments in our first two tasks. This suggests that reading and lexical decision making performance can be dissociated.

## Discussion

The many-to-many model^1–4^ proposes that specialised regions in the brain that recognise faces, will also contribute functionally to word recognition. If this were the case, then one would expect that individuals with DP, who show lifelong impairments of face recognition, to also exhibit deficits in word recognition. In our present study, we administered two lexical decision tasks and five reading aloud tasks to test some of the many-to-many model’s predictions. As a group, our DP participants did not exhibit any deficits in word recognition, thus complementing similar findings in developmental^24, 25^ and acquired cases of prosopagnosia^8, 10, 11^. We therefore propose that face recognition abilities are reliant upon dissociable neural substrates from those involved in reading or lexical decision making; a domain-specific account of face recognition fits best with the data from our present study.

### Impact of Perceptual Information

Our first tasks were designed to test perceptual processing of words by varying word length while controlling for a variety of linguistic factors. Whereas alexia cases will exhibit impairment when speaking longer words aloud due to increasing confusability^26^, our DP cases appear spared in this respect. Similarly, our DP cases exhibited no evidence of impairment when confusability was controlled for on our other tasks. Our results neatly complement similar findings in developmental^24, 25^ and acquired^3^ prosopagnosia cases. The obvious conclusion that seems apparent from these results is that the many-to-many model is fundamentally wrong in its assumption that prosopagnosia cases must share common perceptual deficits in word processing. Instead, a dissociation account for words and faces is more compatible with our current findings.

It should be noted that our DP cases did exhibit elevated error related WLEs when reading words that were matched for average letter confusability. While elevated WLEs are a classic symptom of alexia, these abnormalities should also be apparent in a reading aloud condition uncontrolled for confusability; something we did not find here. Our DP cases only produced abnormal WLEs because they were making fewer errors on the lower length words, with the number of errors made to the longest words in each condition comparable to the controls. This would indicate that DP cases are not actually impaired in reading as their error rates might level out at words over 6-letters long.

### Impact of Linguistic Information

Our second set of tasks examined linguistic processing of words in DP. As in the case of word processing under various perceptual demands, the DP cases as a group seemed unimpaired. Such a finding indicates that DP is not associated with deficits related to their visual experience with words, as indexed by word frequency. This finding is in contrast to such obvious deficits they have in recognizing highly familiar faces. Similarly, despite N being related to processing in the right hemisphere, our DP cases were again generally spared. When the present findings are taken in combination with recent work in developmental^24, 25^ and acquired^3^ cases, one must conclude that the perceptual and linguistic processing of words during reading and lexical decisions is not reliant upon face processing regions. Instead, such processes must rely upon distinct regions that are not involved in face recognition.

### Dissociation Between Reading Aloud and Lexical Decision Making

Despite those with DP appearing spared in their word processing abilities, we do find that one of our cases exhibited a severe reading impairment with spared lexical decision making. This raises the possibility that a small number of those with DP might also have difficulties when processing words, and that these deficits can dissociate judgments of lexicality and reading ability. Prior work on neuropsychological populations has indicated that word naming and lexical decisions can under certain conditions be doubly dissociated^51, 52^, although this is the first time we believe such a dissociation has been shown in DP. The fact that this case is impaired regardless of confusability suggests that their difficulties are distinctly different from those observed in alexia.

Why does this case show reading deficits? Nothing from their neuropsychological profile (Fig. 1) appears to mark them out as being particularly unique from our other DP participants. One explanation might be that they suffer from a global neural abnormality that not only affects their face selective regions, but also their word processing areas’ functioning too. Similarly, it might not be the regions specialized to process words themselves that are abnormal, but rather that there are impairments in these regions’ abilities to route signals to one another. DP cases have been shown to exhibit reduced connectivity from posterior to anterior face selective regions^18, 53^; it might be possible that this DP case also suffers from reduced connectivity between posterior and anterior word selective regions. A third explanation might be that they are impaired at a stage where face and word processing are reliant upon shared cortical resources, possibly in the areas involved in low level visual processing. This latter suggestion seems unlikely though due to the fact that this case is unimpaired in making lexical decisions, and such a task would surely be reliant upon the same low level processes. Neuroimaging research should help elucidate the neural locus of these deficits in such cases.

### Can the Many-to-Many Model be Salvaged?

Our findings are unequivocally incompatible with the many-to-many model’s specific prediction that prosopagnosia cases must be impaired in word processing^4^. Despite this, there are a remarkable number of ways in which data from any word processing experiment in DP can be reinterpreted so that the many-to-many model appears salvageable; or at least salvageable with regards to face and word processing competing for resources in the word and face perception areas of the cortex. For example, if DP cases exhibited superior performance, then this could be interpreted as being due to the existence of domain general, neuronal populations that are typically shared between words and faces during early perceptual processing. The lack of competition in these areas from faces in DP might lead to enhanced cortical specialization for words instead. This greater availability of cognitive resources that undergo ‘neuronal recycling’^54, 55^ would enable DP cases to exhibit superior performance when processing words. Similarly, despite it seeming implausible, unimpaired word processing may only arise due to neuronal recycling counterbalancing similar levels of impairment caused by underlying neural abnormalities. Conversely, any signs of deficits can be interpreted as confirming the many-to-many model’s predictions of prosopagnosia cases being abnormal in word processing due to associative face processing deficits.

All of these interpretations, however, only subserve to highlight an inherent weakness in the many-to-many model’s construction, whereby every single research outcome from DP cases can be interpreted as supporting it. Arguably, one of the most important requirements for a new model is to allow researchers to test very specific hypotheses derived from its predictions. Unfortunately, the many-to-many model in its current form does not allow any such clear predictions to be made. The many-to-many model is exceptional in its simplicity, but such simplicity fails to explain not only why face and word recognition appear dissociable, but also why many aspects of visual word recognition itself can be dissociated into distinct streams that do not overlap. For example, as mentioned earlier damage to the VWFA spares lexical decision making^30^ and also reading under a variety of conditions^26^. Such results are clearly incompatible with the many-to-many model’s prediction that the visual recognition system is functionally integrated. Similarly, the lack of deficits in our DP cases’ response times or error rates suggest that it is unlikely that DP cases to also suffer from subtle abnormalities in their VWFA. Instead, the simple suggestion that word and face processing are dissociable seems to fit best with our findings and recent work^24, 25^.

One possibility that can reconcile the many-to-many model with our data is that face recognition deficits in DP might not be due to impaired processing in perceptual areas, such as the FFA. Instead, these deficits may be caused by regions that process or store face identity information downstream from visual perception. Support for this suggestion comes from DP cases exhibiting atypical memory related electrophysiological responses, which are thought to be driven by activity in the hippocampal area^45^, and abnormalities in their anterior temporal lobe^56^. However, there appears sufficient neuroimaging evidence to suggest that abnormalities in the occipital and fusiform face areas are at least in part causing face recognition problems in DP^15, 18, 19, 57^. Similarly, even our DP cases with severe deficits in perceptual processing, as indexed by the CFPT, do not seem to exhibit any greater difficulty processing words than our non-apperceptive cases; the one DP case who was impaired when reading, also displayed no related perceptual deficits in face processing. Taken together, it would seem that perceptual difficulties in processing faces do not produce associative impairments when processing words.

In conclusion, we have found no evidence that individuals with DP are impaired when recognizing words. Our findings are incompatible with the suggestion that deficits in face recognition must also lead to word processing impairment^4^. While a number of arguments have been made against the suggestion that face recognition is not distinct from other forms of visual recognition^1, 58, 59^, we again find compelling evidence that such an ability must rely upon dissociable processes from that of word recognition. A modular view of face processing is supported by a wealth of animal^60^, infant^61–63^, behavioural^64, 65^, genetic^66^, neuroimaging^67–69^ and neuropsychological^8, 10, 70^ research. The present study contributes to this already ample body of work that face recognition in adulthood is domain-specific and does not overlap with reading or lexical decision making.

## Electronic supplementary material

Supplementary Information
