# Supplementary material for: Intact word processing in developmental prosopagnosia
Source: Sci Rep. 2017 May 10;7:1683. doi: 10.1038/s41598-017-01917-8 (PMC5431912; doi:10.1038/s41598-017-01917-8)
Supplement: Supplementary file 1 — Supplementary Information [file 41598_2017_1917_MOESM1_ESM.doc]

**Intact word processing in developmental prosopagnosia (Supplementary Information)**

Edwin J. Burns^1^, Rachel J. Bennetts^2^, Sarah Bate^2^, Victoria C. Wright^3^, Christoph T. Weidemann^4,5^ & Jeremy J. Tree^4^.

^1^Nanyang Technological University

^2^Bournemouth University

^3^Aberystwyth University

^4^Swansea University

^5^University of Pennsylvania

Bayesian analyses allow us to make inferences as to how likely our data fits with the null or alternative hypotheses. In this respect, they seem like an ideal way of examining whether DP cases are either similar, or different, in their performance from controls when processing words. Here we report BF_10_ in all of our Bayesian analyses: while any value of BF_10_  > 1 could indicate support for the alternative hypothesis, only BF_10_  > 3 would generally be considered as positive evidence (for a detailed discussion regarding Bayes Factors, see Jarosz & Wiley, 2014). Conversely, any value of BF_10_  < 1 would indicate support for the null hypothesis; i.e., there are no differences between our DP cases and controls. The model which has the strongest evidence as indicated by BF is preferred over other models. All Bayesian analyses were performed using JASP (Love et al., 2015).

*Impact of Perceptual Information*

*Lexical Decision: Length (word confusability not controlled)*

To test for any possible effects of lexicality between the groups, response times were subjected to a mixed model Bayesian ANOVA with Stimuli (words vs. non-words) as a within subject factor and with Group (controls vs. DP) as a between subject factor. These analyses showed that the Effects model [BF_10_ = 179] was preferred over the Group [BF_10_ = .56] and the Group x Effects models [BF_10_ = 107]. The same analyses on the errors also supported the Effects model [BF_10_ = 152241] over the Group [BF_10_ = .75] and Group x Effects models [BF_10_ = 62152].

To examine any possible differences between the groups across word length, we performed a mixed model Bayesian ANOVA with Length (3 vs. 5 vs. 7 letters long) as a within subjects factor and Group (controls vs. DP) as a between participants factor. These analyses showed that the Group model [BF_10_ = 1.24] was preferred over the Effects [BF_10_ = .35] and Group x Effects models [BF_10_= .34], although it should be noted that there was no strong evidence for differences between our participant groups. The same analyses on the errors supported the Effects model [BF_10_ = 1.36^+11^] over the Group [BF_10_ = .52] and Group x Effects model [BF_10_ = 8.18^+10^]. Bayesian independent t-tests on the word length effects for the response times and errors similarly revealed no differences between the groups [BF_10_ = .65 and .53 respectively].

*Reading Aloud: Length (word confusability not controlled)*

We performed a mixed model Bayesian ANOVA with Length (3 vs. 5 vs. 7 letters long) as a within subjects factor and Group (controls vs. DP) as a between participants factor on the response times. The effects model [BF_10_ = 11.3] was preferred to the Group [BF_10_ = .69] and Group x Effects [BF_10_ = 8.04] models. Similar analyses on the errors found no evidence to support either the effects or interaction models over the null hypothesis [BF_10_ < 1]. Between samples Bayesian t-tests revealed no differences between the groups in their response time or error related word length effects [BF_10_ = .36 and .5 respectively].

*Reading Aloud: Length (sum confusability maintained across words)*

A mixed model Bayesian ANOVA with Length (3 vs. 5 vs. 7 letters long) as a within subjects factor and Group (controls vs. DP) as a between participants factor on the response times supported the Effects model [BF_10_ = 65435] over the Group [BF_10_ = .74] and Group x Effects [BF_10_ = 48524] models. The same analyses on the errors showed a preference for the Effects model [BF_10_ = 4.48] over the Group [BF_10_ = .69] and Group x Effects [BF_10_ = 2.99] models. Bayesian comparisons showed no evidence of differences between the groups in either their response time or error related WLE [BF_10_ = .35 and .9 respectively].

*Reading Aloud: Length (average letter confusability maintained across words)*

A mixed model Bayesian ANOVA with Length (3 vs. 5 vs. 7 letters long) as a within subjects factor and Group (controls vs. DP) as a between participants factor on the response times supported the Effects model [BF_10_ = 7.28^+7^] over the Group [BF_10_ = .7] and Group x Effects [BF_10_ = 4.86^+6^] models. Similar analyses on the errors produced no evidence for the Effects [BF_10_ = .29], Group [BF_10_ = .51], or Group x Effects [BF_10_ = .15] models. Between group Bayesian comparisons showed no strong evidence for the response time and error related WLE being different between the groups [BF_10_ = .48 and 1.2 respectively].

*Impact of Linguistic Information*

*Lexical Decision: Frequency x Age of Acquisition (AoA)*

To test for any possible effects of lexicality between the groups, response times were subjected to a mixed model Bayesian ANOVA with Stimuli (words vs. non-words) as a within subject factor and with Group (controls vs. DP) as a between subject factor. The Effects model [BF_10_ = 752] was preferred to the Group [BF_10_ = .74] and Group x Effects [BF_10_ = 693] models. A similar ANOVA performed on the errors also revealed that the Effects model [BF_10_ = 1.85] was preferred to the Group [BF_10_ = .48] and Group x Effects [BF_10_ = 1.06] models.

To examine any possible differences between the groups across the linguistic conditions, we performed a mixed model Bayesian ANOVA with AoA (early vs. late) and Frequency (high vs. low) as within subject factors and Group (controls vs. DP) as a between participants factor on the response times. These analyses revealed that the Frequency + AoA model [BF_10_ = 26761] was preferred to all other models involving Group [all BF_10_ < 15046]. Similar analyses on the errors revealed the same finding, with the Frequency + AoA model [BF_10_ = 329253] preferred over the other models involving Group [all BF_10_ < 278701].

*Reading Aloud: Frequency x AoA*

To examine any possible differences between the groups when reading words across the different conditions, we performed a mixed model Bayesian ANOVA with AoA (early vs. late) and Frequency (high vs. low) as within subject factors and Group (controls vs. DP) as a between participants factor. These analyses showed no evidence for any main effects or interactions [all BF_10_ < 1]. The same ANOVA performed on the errors yielded the same results [all BF_10_ < 1].

*Reading Aloud: N Confusability*

To examine any possible differences between the groups when reading words varied by confusability and N, we performed a mixed model Bayesian ANOVA with Confusability (high vs. low) and N (high vs. low) as within subject factors and Group (controls vs. DP) as a between participants factor. We found evidence in support of the N + Confusability effects model [BF_10_ = 88] over all other models involving Group [all BF_10_ < 65]. Similar analyses on the errors revealed no evidence for any alternative hypothesis models [all BF_10_ < .64].

*Global Analyses of Behavioural Tasks*

As with our Frequentist analyses, we were curious whether our Bayesian analyses would reveal any evidence for differences between our DP cases and controls across all the tasks. We therefore performed a 2 x 2 x 7 mixed model ANOVA on our participants’ z-scores, with a between subject factor of Group (controls vs. DP) and within subject factors of Experiment (1, 2, 3, 4, 5, 6 and 7) and Measure (Response Times and Errors). As with the Frequentist analyses in the main manuscript, these analyses revealed no evidence for performance differences between our DP cases and controls, instead showing support for the null hypothesis [BF_10_ = .46]. In sum, a comprehensive battery of 7 experiments revealed no evidence to suggest DP cases are in any way different in processing lexical stimuli.

*Word Length Effects for Individual DP Cases*

| **Participants** | **Age** | **Sex** | **LDT**  **Not Cont**  **(ms/l)** | **Naming  Not Cont**  **(ms/l)** | **Naming**  **Sum Cont**  **(ms/l)** | **Naming**  **Ave Lett**  **(ms/l)** |
| --- | --- | --- | --- | --- | --- | --- |
| DP1 | 32 | M | -0.69 | -7.67 | 8.55 | 56.9 |
| DP2 | 21 | F | 6.08 | -7.98 | -4.08 | 8.25 |
| DP3 | 32 | M | -5.2 | -1.53 | -3.53 | 12.05 |
| DP4 | 32 | F | NA | NA | NA | NA |
| DP5 | 22 | F | -35.86 | 28.12 | 53.7 | 7.71 |
| DP6 | 20 | F | -53.3 | -3.99 | 18.02 | 4.01 |
| DP7 | 73 | F | -4.47 | 7.07 | 3.95 | 42.51 |
| DP8 | 53 | F | -54.87 | 57.23 | 49.17 | 45.93 |
| DP9 | 56 | F | 47.97 | 40.45 | 26.33 | 42.63 |
| DP10 | 64 | M | -28.57 | 16.16 | 37.03 | 1.78 |
| DP11 | 52 | F | -35.47 | 18.49 | 14.19 | 32.33 |

**Table 1**. **Response time related word length effects for each DP case per task.** Task columns indicate from left to right: lexical decision task word confusability not controlled (LDT Not Cont), naming task word confusability no controlled (Naming Not Cont), naming task sum confusability maintained across words of different lengths (Naming Sum Cont) and naming task where average letter confusability was maintained across words of different lengths (Naming Ave Lett). DP4 did not participate in the length tasks. The values indicate ms/letter.

| **Participants** | **Age** | **Sex** | **LDT**  **Not Cont**  **(errors/l)** | **Naming  Not Cont**  **(errors/l)** | **Naming**  **Sum Cont**  **(errors/l)** | **Naming**  **Ave Lett**  **(errors/l)** |
| --- | --- | --- | --- | --- | --- | --- |
| DP1 | 32 | M | -0.5 | 0.5 | 0.5 | 0.5 |
| DP2 | 21 | F | 0 | 0 | 1 | 0 |
| DP3 | 32 | M | -1 | 0 | 1 | 0.5 |
| DP4 | 32 | F | NA | NA | NA | NA |
| DP5 | 22 | F | 0 | 0.5 | 0.5 | 0 |
| DP6 | 20 | F | -2 | 0 | 0.5 | 1.5 |
| DP7 | 73 | F | 1 | 0 | 0 | 1 |
| DP8 | 53 | F | -2 | 0 | 0 | 0 |
| DP9 | 56 | F | -1 | -0.5 | 1 | 0.5 |
| DP10 | 64 | M | -1 | 0 | -0.5 | 0.5 |
| DP11 | 52 | F | -1 | -1 | 1.5 | 1.5 |

**Table 2**. **Error related word length effects for each DP case per task.** Columns indicate from left to right: lexical decision task word confusability not controlled (LDT Not Cont), naming task word confusability no controlled (Naming Not Cont), naming task sum confusability maintained across words of different lengths (Naming Sum Cont) and naming task where average letter confusability was maintained across words of different lengths (Naming Ave Lett). DP4 did not participate in the length tasks. The values indicate errors/letter.
